# A fully automatic apparatus for chemical reactions on the laboratory scale

**DOI:** 10.1155/S1463924685000074

**Published:** 1985

**Authors:** M. Legrand, P. Bolla

**Affiliations:** Direction des Services Scientifiques Généraux Roussel Uclaf 102 Rte de Noisy Romainville F93230 France


Journal of Automatic Chemistry, Volume 7, Number 1 (January–March 1985), pages 31-37

### A fully automatic apparatus for chemical reactions on the laboratory scale

**M. Legrand and P. Bolla**

*Direction des Services Scientifiques Généraux, Roussel Uclaf, 102 Rte de Noisy, F93230 Romainville, France*

### Introduction

The apparatus described here is designed to automatically perform common chemical reactions on some tens to hundreds of grams of reactants. Its principal application is the necessary optimization of parameters when going from the research-laboratory scale to the pilot plant in fine organic chemistry, an operation which requires numerous repetitive assays [1 and 2].

It is also useful for the preparation of a given quantity of products simply by repeating the same reaction when scaling up is too difficult, or time-consuming. Moreover, the physical presence of the chemist near the apparatus is not necessary, which is helpful when hazardous reactions have to be studied. Furthermore, the strict control of many parameters, and the possibility of sampling the reaction medium during the course of the operation, means that information about the kinetics of the reaction can be collected.

These considerations are important to all companies involved in fine organic chemistry (especially pharmaceutical firms); and several studies about automation at the laboratory scale have recently been published [3, 4 and 5].

An important characteristic of the authors' system is its versatility – it can carry out most of the chemical reactions currently used in organic chemistry, and its software allows user-friendly interactions between chemists and computer.

The system involves three parts: the chemical set, the computer and interfaces, and the software.

### The chemical set

Figure 1 shows the main parts of the apparatus, and figure 2 is a partial schematic description of the chemical system. The reactor is of a classical design and is equipped with a stirrer and a cooler, including a splitter which allows either a reflux or a distillation of the solvent, depending on the state of an electromagnetic valve (not drawn on figure 2).

The heating is electrical, by means of a resistive silver coating applied to the bottom of the reactor, and the cooling is obtained by immersion of the reactor into a cooling-bath placed on a pneumatic jack.

The system is fully automatic and all transfers are performed by the machine; most of them are quantified at the same time. A transfer for solid or liquid reagents involves two steps. First, the product is brought into an intermediate vessel linked to a strain-gauge for weighing and then transferred to the reactor (see figure 3 – an example with powder). The driving force for transfer is obtained by vibration (powders) or pumping (liquids).

There is an additional container to allow solvents to be used for rinsing – pressure is applied to dispatch them to different parts of the apparatus.

For introducing small quantities of liquid over a long period of time, peristaltic pumps are preferred. Non-quantified introductions are also possible, and are generally used for neutralization under the control of such a parameter as pH or redox.

At the present time, the set can accommodate four solid reactants, seven liquid reagents and four solvents. All

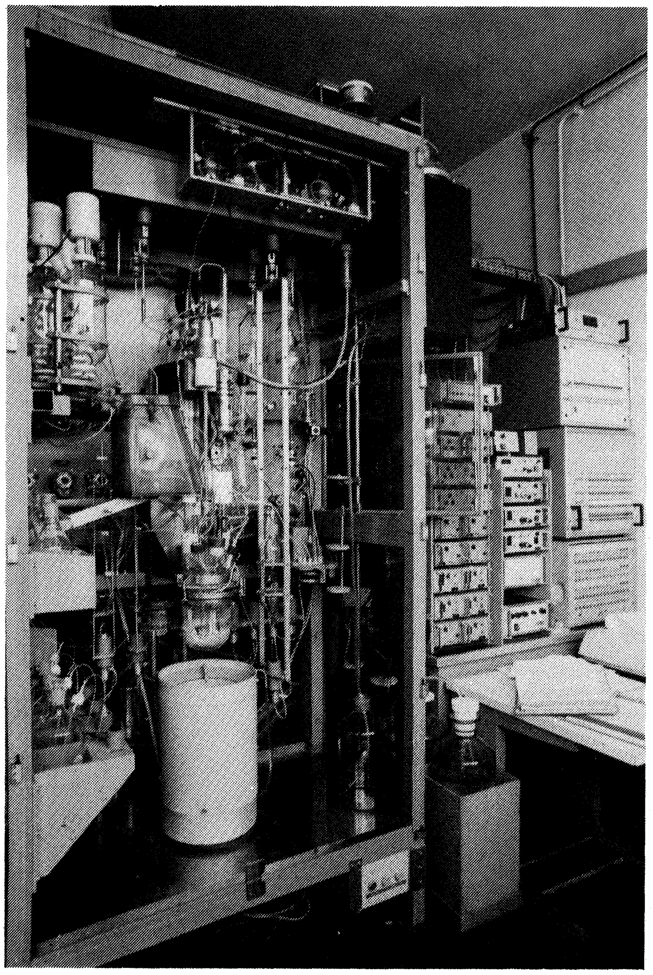

*Figure 1. General view of the apparatus.*

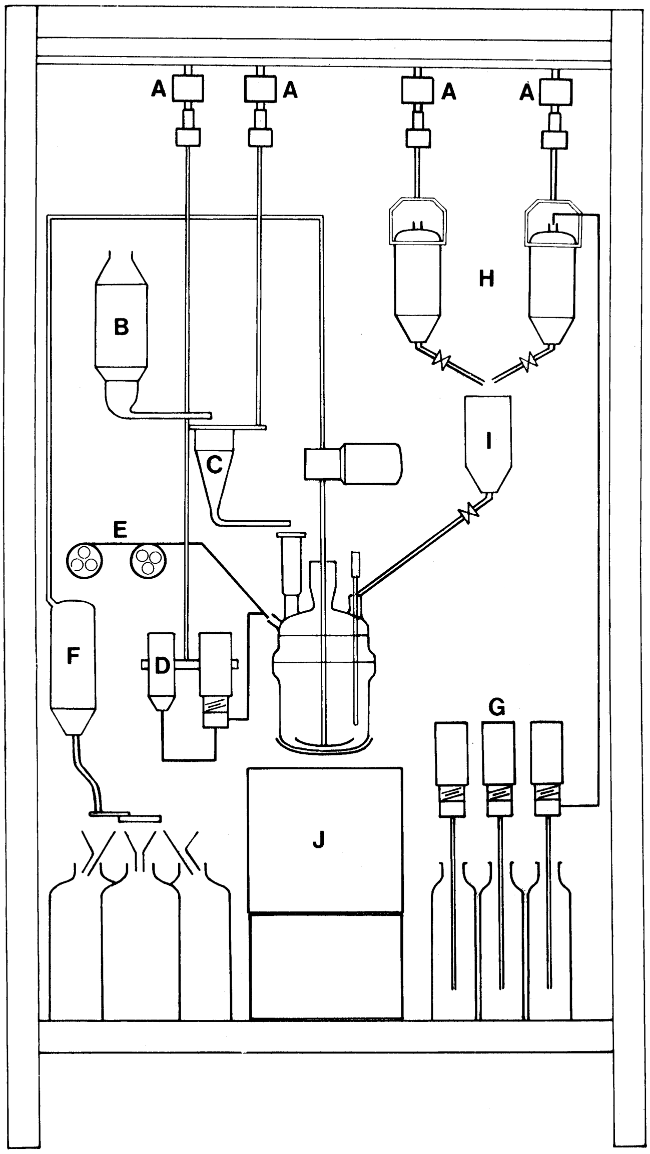

*Figure 2. Schematic description of the main parts of the chemical system. Where A = strain gauges; B = powder dispenser; C = weighing vessel for powders; D = weighing vessel for liquid reagent with its transfer pump; E = peristaltic pumps; F = recuperation of the reaction mixture; G, H and I = solvent weighing and distribution; and J = pneumatic jack for cooling.*

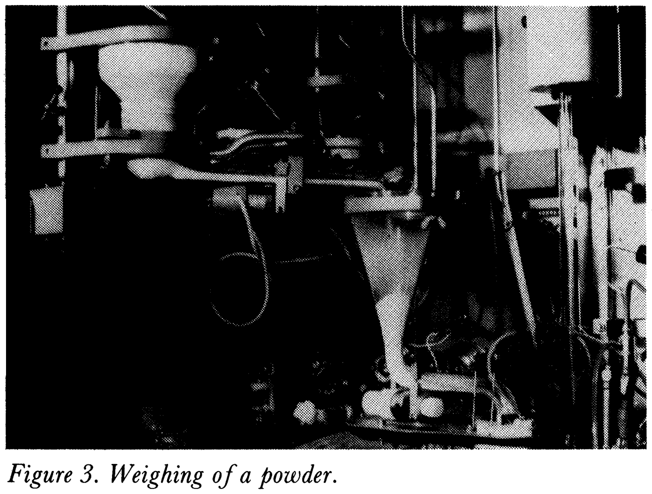

*Figure 3. Weighing of a powder.*

introductions can be monitored in relation to the state of the reaction.

At the end of the chemical reaction, the reaction mixture is transferred to one of six flasks through an intermediate container and a distributor. Vacuum is used for the first transfer and the content of the reactors is flushed through the hollow axis of the stirrer. The second transfer is obtained by gravity.

Several rinsings are usually carried out – they are either added to the reaction mixture or sent to the drain by appropriate positioning of the outlet. Finally, a general washing is performed and the different components are dried by a stream of hot nitrogen. The set is then ready for a new run.

A sampling system, applying principles used in analytical laboratories (figure 4), provides a means of automatically and quantitatively selecting samples for further analysis. It consists of a hairpin-shaped tube immersed in the reacting mixture. One branch of the tube can be separated in two parts by a pneumatic jack. When open, a peristaltic pump samples a given volume of the liquid. Then the U-tube is closed and the sample is washed out by the solvent.

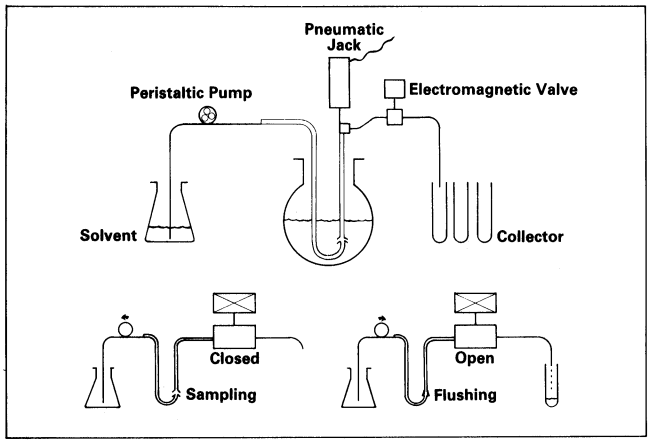

*Figure 4. Sampling system.*

Up to 10 analogue probes can be connected to the system. In most cases, only five are used to measure the temperature of the reactor, the temperature of the cooling-bath, the temperature at the entrance of the cooler, pH and redox potential.

Most of the sensors and actuators are part of machines widely used in the authors' research centre and have already been reported [7].

As certain reactions require intermediate decantation of the reactive mixture, an automatic device has been recently added to do this (the device is not shown in any of the figures in this paper).

### Computer and interface

The system is controlled by a DEC PDP 11/10 computer with 28 K words of central memory and two RKO5

cartridge disks of 1.2 mega 16-bit words each, operating under the RT11 foreground/background system, version 2 c.

Figure 5 shows the interfacing. Most of the components seen on the left-hand side of the figure are directly connected to the actuators and allow the execution of orders given by the computer.

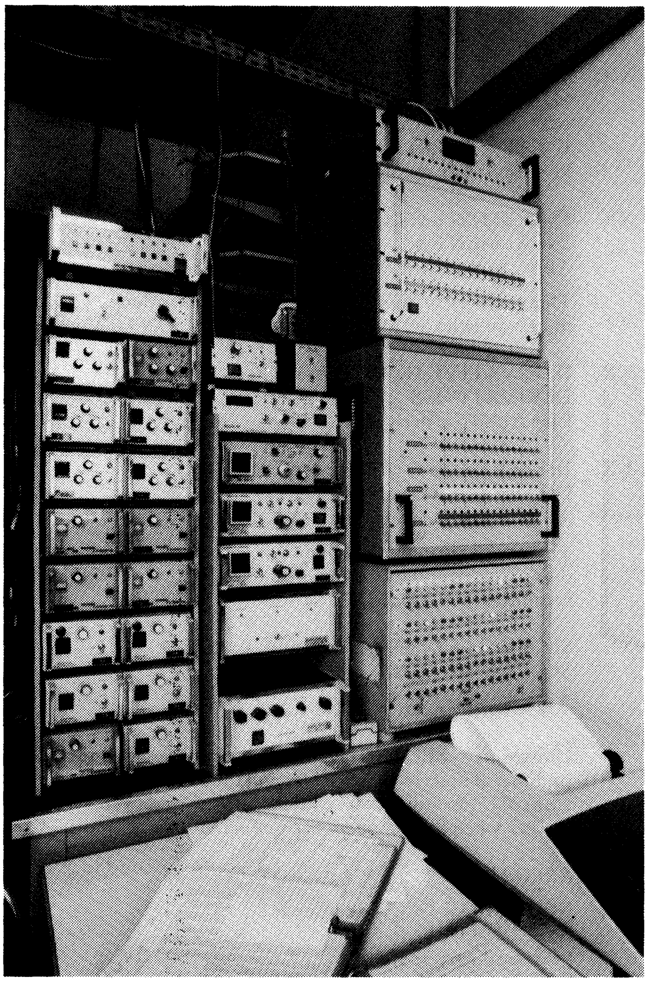

*Figure 5. Interfacing. For explanation see text.*

A 20 000 points analogue/digital (A/D) converter is connected through a multiplexer (16 channels) to the specific interfaces of the sensors.

A command panel, shown on the lower part of the right-hand side of figure 5, allows manual back-up. However, a reaction cannot be carried out by this method because all the control loops need the computer to be active; the manual switch can only be used to transfer the products and to bring the set back to its initial condition after a failure.

### Software

Two objectives were kept in mind during the programming: versatility and simplicity for the chemist.

Versatility has been obtained by a complete independence of the different actions. Every elementary operation – like weighing, transferring, measuring, heating, cooling, counting a time etc. – has a corresponding routine not directly connected to the others. The exchange of information, logical or numerical, is possible through tables. It must be noted that in regulation loops only on-off control is used and three separate routines correspond to the three steps normally encountered in such a loop: that is measuring, comparing and actuating. Consequently, any actuator can be connected to any sensor through the same routine of comparison, which adds to the versatility of the system.

On the other hand, a table of actions contains the addresses of the routines which may or may not be flagged. Every half-second, this table is examined and only the flagged routines are executed. The flagging of the right routines at the right time is obtained through a protocol. (A protocol is a sequence of steps, each of which contains the code for the elementary action to be performed and several arguments for the transfer of the necessary information.)

Every half second, an internal routine tries to take into account a new step but can only do it if the state of the apparatus allows it. This regulation is necessary because some operations can be performed simultaneously, but others cannot: for instance a reactant cannot be weighed and transferred at the same time.

Most of the interdictions are dependent on the apparatus itself, as is the example given above, and they are permanently present in the corresponding statement. However, the chemist can introduce interdictions of his own for controlling the sequence of operations to any degree of complexity.

During a run, the protocol is stored on a disk and read, block-by-block, during the course of the reaction.

The protocol language, which can be understood by the machine, is practically undecipherable by the chemist. Therefore a language has been designed, which uses, as far as possible, expressions common to chemists. The training necessary for its use is minimal; it can often be understood, without training.

The language uses two types of statements: the first type is *declarations* which indicate the location of reagents in the different containers (figure 6 provides some examples of the syntax of the declarations). On the left side of the sign '=', the chemist can use any word he likes. On the right-hand side there are a couple of codes separated by a semicolon, characterizing the actuators which transfer the specified product from its container to the reactor through the weighing vessel; these codes are fixed. In the protocol, when necessary, the different products participating in the reaction are named by the word that the chemist has chosen in the declaration.

The second type of statement corresponds to *instructions* given to the machine to execute operations. One step of the protocol corresponds to one instruction (figure 6); the general syntax is as follows. XX is an integer for numbering the lines of the program. The part of the instruction, named 'operation' in figure 6, specifies the type of action wanted: for instance 'stirring, weighing etc.'. Self-explanatory terms are used, as far as possible, in such a way that a protocol can be understood with a minimum of training. The name of the action can be followed by arguments, the number of which depends on the nature of the action. Finally, the chemist may add interdictions so that the step cannot be allowed to proceed if the apparatus is not ready for it. For instance in figure 6, 'Eyy' means that the statement will become active only when a preceding step, 'yy', is completed. 'Tj' corresponds to a chronometer started by a preceding instruction: the instruction 'XX' will become active only when the time declared by the chronometer is over. Lastly, 'Lk' is a logical variable also declared in a preceding instruction. If this option is used, the instruction 'XX' will be taken into account the first time the logical variable takes the value 1.

```
DECLARATIONS
      BR = R1 ; I1
      H20 = P4 ; SL
      ETOH = P2 ; SM
      PC15 = V3 ; VP

                    STATEMENTS
XX,   OPERATION [(Arguments)] [Eyy] [,Tj] [,Lk].
```

*Figure 6. Examples of the syntax of declarations.*

The operations to which the second type of instruction refers can be classified in four groups: measurements, actions, logical operations and utilities.

*Measurement*

Figure 7 illustrates three types of measurement. All corresponding instructions are followed by the letters 'G' (for Go) or S (for Stop) because a measurement is continuous; it must be started and then stopped when no longer necessary.

When in operation, measurements (or computations) are made every half-second and the new values replace the old ones in internal tables.

Analogue measurements are activated by the statement 'SENSOR-G'. The arguments characterize the sensors which must be put into action (or stopped). Presently there are five possibilities: temperature of the reactor, of the reflux, of the cooling bath, pH and redox, but there is room for six more if necessary.

A second type of measurement, less common to chemists, is one the authors' call 'digital measurement'. It is the integrated number of actions of actuators versus time. When the actuator is controlled by a variable characteristic of the reaction, this measurement is in close relation with the kinetics of the process; it explains the name of the statement. In figure 7, the codes given correspond to the more relevant actuators of the system, pumps, cooling and heating.

*ANALOGICAL*

XX, SENSOR-G (code 1 [,code 2 . .])

SENSOR-S

Codes : TR, TX, TF, PH, RX

*DIGITAL*

XX, KINETICS-G (code 1 [,code 2 . .])
KINETICS-S
Codes : X1, X2, CC, CR, CA, CB, CP

*COMPUTED*

XX, SHIFT-G (AB : Sensor, Cte; . . .)
SHIFT-S
AB = Sensor (t)-Cte
IF CTE = ∅ AB(t) = Sensor(t) − Sensor(to)
to = time when the statement is taken into account

XX, INCREMENT-G (AB: Sensor, Cte; . . .)
INCREMENT-S
$AB = (Sensor(t_j) - Sensor\ (t_{j-1}))^* Cte + Sensor(t_j)$
These two instructions are often used in control loops when a more sophisticated algorithm than a simple 'ON-OFF' is desired

XX, DERIVATIVE-G (AB : Sensor, Cte; . . .)

DERIVATIVE-S

Cte : Δt, step of derivation

XX, DIFFERENCE-G (AB: Sensor, Cte; . . .)
DIFFERENCE-S
AB = Sensor(t) − Sensor(t − Δt)
These two instructions have similar aims; the latter is used when the derivative of the function is small and of the order of magnitude of the noise.

XX, SLOPE-G (AB: Sensor, final value, units, time)
SLOPE-S
$$AB = Sensor(t_o) + \frac{units}{time} * (t\text{–}to)$$
Units/time = SLOPE. It is easier to give successively the range (number of units of the variable) and the corresponding time Ex. Units 5° C (for) time 25 s.
to: time when the statement become active

*RECORDING*

XX, NOTEBOOK-G (t, code 1 [,code 2, . . .])
NOTEBOOK-S
t = recording period
Code = code of analogic, digital or computed measurement.

[ ] optional argument.

*Figure 7. Three types of measurement.*

The third type is computed measurements: that is measurements resulting from the combination of analogue, digital and possibly other computed measurements. At the moment, only five possibilities are offered – 'SHIFT', 'DERIVATIVE', 'DIFFERENCE', 'INCREMENT' and 'SLOPE'. Others can be added if necessary, but some programming would be required.

The computations that these instructions perform need not be described in full – they are outlined in figure 7.

Closely related to the measurements is the statement 'NOTEBOOK', which allow storage of the data on the disk. As it is generally not necessary to record data every half-second, at least not for all phases of a reaction, the period can be chosen by the chemist.

*Actions*

The instructions of this class concern the actuators. The action can be continuous. In that case, the statements are paired and are characterized by the letters 'G' and 'S'. Some are shown in figure 8.

```
XX,  STIRRING-G (v)
     STIRRING-S
     v = 1 to 4 defines one of four speeds

XX,  ACTUATE-G (code: Li)
     ACTUATE-A (code)
     code = code actuator
     Li = Logical variable

XX,  THERMO-G (Li, Lj)
     THERMO-S (Li, Lj)
     Li = logical variable, heating
     Lj = logical variable, cooling
     (Li, Ø) heating only
     (Ø, Lj) cooling only.
```

*Figure 8. Continuous actions.*

'STIRRING' switches the stirrer on and controls the speed.

'ACTUATE' energizes the actuators, the code of which is given as an argument if, and only if, the logical variable is one. This variable is defined by a special statement described in the next paragraph.

'THERMO' controls the heating and the cooling of the reactor. Of course, 'Li' and 'Lj' must not take the value '1' simultaneously. If they do, an error signal appears which stops the operation.

The other actions are discontinuous (Examples of statements related to some of them are illustrated in figure 9). The first two are used for quantitative transfer of reagents:

'WEIGHING', takes the product to the weighing container and 'ADDING' transfers it into the reactor.

'ADDING' has optional arguments which allow very fine control of the operation. For instance, if the introduction of the reagents must be done in several fractions in a given time, the argument 'FRn' is used, where 'n' is the number of fractions, accompanied by the logical variable 'Tj'. The latter characterizes the period of the introduction and is defined in a special statement.

'Lk' is a logical variable; if it is equal to zero, the transfer is stopped; it restarts when 'Lk' takes value 1 again. In other words, the introduction can be put under the control of different variables – 'T', 'pH', 'RX' or a combination of these. 'ADn' is also a logical variable, but its action is different; the first time 'ADn' takes the value zero, the introduction is definitely stopped, even if the indicated quantity has not been totally introduced. With this statement, practically all the conditions normally met in chemistry for the quantitative transfer of reactants can be solved.

At the bottom of figure 9, for instance, the necessary statements for introducing 100 g of alcohol in the reactor are explained. The operation is easy to understand.

```
XX,  WEIGHING (Name: weight)
XX,  ADDING (Name: weight [,FRn, Tj, Lk, ADn])
     Name is the name of the product declared previously

XX,  VOLUME (Name: volume [,t, Ln, ADn])
XX,  SAMPLING (VP: vol 1, VR: vol 2 [,period, n, Li])
XX,  TRANSFER (Code)
XX,  DRAW-OFF
XX,  STORE
XX,  TANK-A
XX,  DRYING-C [(code)]
XX,  DRYING-H [(code)]

     ETOH = P1 ; SM
10,  WEIGHING (ETOH : 100)
20,  ADDING (ETOH : 100)
30,  TRANSFER (RR).
```

*Figure 9. Discontinuous actions.*

In this special case, the same quantity is weighed, then transferred. This is not compulsory: the introduction can be made in several steps, provided that the weighed quantity is not smaller than the sum of the introduced quantities.

'Volume' concerns the quantitative introduction of liquids by peristaltic pumps. Here, the optional argument 't' defines the total time allocated to the introduction of the given volume. If omitted, the introduction is done at the highest speed.

'SAMPLING' governs the sampling of a given volume of the reacting mixture for analysis.

'TRANSFER' is used for solvents.

'DRAW-OFF', 'STORE' and 'TANK-A' control the transfer of the reacting mixture into one of the collector's flasks.

'DRYING' is for the drying of the set after cleaning. 'C' means 'Cold' (cold nitrogen is used), and 'H' is for 'Hot'.

*Logical operation*

Sequential operations as well as control loop are of the 'on–off' type. So logical variables and the means of computing them are important.

'LOGIC' allows the computation of more or less complex Boolean expressions. The chemist has at his disposal up to 16 logical variables ('L1' to 'L16') which he can define as indicated here. Figure 10 is an example of the syntax used.

'CHRONO' is used when time is an explicit parameter. 'Ti', where 'i' can take the values 1 to 4, is a logical variable which is zero when the statement is taken into account by the program and becomes 1

when the time 't' has elapsed. The optional argument allows the operation to be repeated 'r' times.

'STABILITY' is a more specific statement.

'Li' takes the value 1 when another logical variable 'Lj' defined previously, keeps on the value 1 for at least 'n' seconds. (The optional slash inverses the proposition, that is 'Li' is one when 'Lj' remains at zero for at least 'n' seconds.) This statement is used to define the end of an operation: a neutralization for instance. Close to the equivalence point, the pH oscillates around the threshold value at each drop of the reagent. The reaction is considered finished when the period of these oscillations becomes longer than a given value. This statement allows this operation.

In the automatic set described here, control loops are explicitly detailed: that is sensor, comparator and actuator are related to specific statements. For instance, the introduction of a reagent under the control of pH and temperature corresponds to the partial protocol shown in figure 10. First, sensors for temperature and pH are activated, then the logical value 'L1' (corresponding to the Boolean expression) is computed every half-second from the refreshed value of 'TR' and pH, and finally the acid is pumped into the reactor when 'L1' is 1.

```
XX,   LOGIC (L1 : exp 1 [;L2 : exp 2; . . . ])
XX,   CHRONO (Ti:t [,r])
XX,   STABILITY (Li: [/] Lj, n)

10,   SENSOR-G (TR,PH)

 .

 .

30,   LOGIC (L1:TR<50*AND*PH>6)

 .

 .

60,   ACTUATE-G (PA:L1).

```

*Figure 10. Logical statements.*

*Utilities*

'CLOSE' stops all the continuous actions.
'STOP' terminates the protocol.
'WAIT' momentarily closes the protocol.

This last statement allows the chemist to deliver discrete orders to the set. When one statement, or a group of them, is followed by 'WAIT', the machine executes it and remains in the corresponding final state until a new group of statements is given. This semi-automatic type of working is useful when, at the beginning of a study, the chemist is not confident about his experimental procedure. He can then orient the reaction from his observations.

With the 'utilities' statements, practically any procedure compatible with the chemical hardware of the apparatus can be performed.

This language might appear rather sophisticated for such an application, but a principal objective was that this automation be a tool for chemists: easy to use, even occasionally, without the help of a specialist in computer science.

A second objective was that the executives in charge of research groups be able to understand any protocol with practically no training.

Figure 11 shows the beginning of a protocol for studying the hydrolysis of ethyl allylacetylacetate, followed by a condensation with pyruvaldehyde and a decarboxylation. It illustrates the ease with which this language can be followed.

Protocols composed in this way are easy to understand but cannot be directly interpreted by the program monitoring the apparatus. It must be compiled by a special background program. This not only translates the protocol but debugs it and edits diagnostics if syntax or logical errors are met.

| | | |
|---|---|---|
| DECLARATIONS | EAAA = R1 ; I1 | Ethyl allylaeetylacetate |
| | H2O = P2 ; SM | Water |
| | NaOH = M2 ; S2 | Solution of sodium hydroxide |
| | PYA = M1 ; S1 | Pyruvaldehyde |

| | |
|---|---|
| 10 WEIGHING (H20 : 480) | WEIGHING of 480 g of water |
| 20 WEIGHING (EAAA : 150) | WEIGHING of 150 g of ethyl allylacetylacetate |
| 30 ADDING ($H_2O$ : 480) | 480 g of $H_2O$ are transfered in an intermediate vessel |
| 40 TRANSFER (RR) | TRANSFER under pressure of $H_2O$ contained in the intermediate vessel – RR = REACTOR |
| 50 STIRRING-G (2) | Stirrer is put into action, speed 2 |
| 60 SENSOR-G (TR, PH, RX) | Analogical temperature, pH and redox sensors are switched on |
| 70 LOGIC (L1:TR<18; L2:TR>20) | L1 is true (=1) when TR reaction mixture temperature is <18°; L2 true when TR>20° |
| 80 THERMO-G (L1, L2) | When L1 = 1 (TR < 18°) the heater is switched on, when L2 = 1 (TR>20°) the cooling bath is in contact with the reactor |
| 90 ADDING (EAAA : 150) | 150 g of EAAA are transferred in the reactor after weighing |
| 100 THERMO-S E90 | After step B E90 has been performed, temperature regulation is stopped (E90 is one type of interdiction) |
| 110 VOLUME (NaOH : 98, T3600) | 98 ml of a solution of NaOH are introduced through a peristaltic pump over 3600 s. |

*Figure 11. Protocol for studying the hydrolysis of ethyl allylacetylacetate.*

Once compiled, the protocol is stored in an intermediate file. For starting a new run, protocols are concatenated if more than one rection is to be monitored, then the first protocol is started.

At operating time, many tests are carried out and if an anomaly is detected, a diagnostic is issued. Some of them are just warnings, others are fatal, in which case the reaction is stopped.

The cooling device is designed in such a way that if such a condition occurs (even a power failure) the reactor gets immersed in the cooling bath; moreover, a battery-operated device allows the introduction of a neutralizer if necessary.

These features help to keep the system safe, even in the event of a major breakdown.

In addition to the program just described, some 'utilities' routines are used for performing various tasks before, during and after the reaction – they are briefly described in figure 12.

| | |
|---|---|
| | *Used before reaction* |
| STANDARD | Standardization of sensors |
| VERIFY | Verification of sensors |
| PERI | Standardization of peristaltic pumps |
| COEFPA | Manual modification of standardization coefficients |
| | *Used during reaction* |
| LECENC | Editing of parameters measured by sensors in activity |
| COMREA | Introduction of information in the file corresponding to the reaction |
| MODLOG | Modification of thresholds for control loop |
| | *Used after reaction* |
| REAFIN | Introduction of information in the file corresponding to the reaction |
| LABOOK | Editing of information and data stored in the file during the reaction. |

*Figure 12. Utility routines.*

### Conclusion

This set has been in operation for three years and is now totally reliable.

It has been well accepted by the chemists who rapidly became expert in the use of the language for writing protocols and who now apply entirely new procedures without any prior testing in the laboratory, which shows the versatility of the system.

Reproducibility is generally good, of the order of magnitude of the analytical error, that is 1 to 1.5% over a period of several months.

At the moment, the set's most striking advantage is certainly an important gain in time, especially for reactions lasting more than 8 to 10 h and requiring intricate procedures. Furthermore, a chemist's time previously spent in surveying can now be devoted to more creative work, for instance studying new synthetic pathways or designing procedures for optimization, as a result of which the productivity of the laboratory is increased.

Furthermore, the various possibilities offered by the set of instructions allow sophisticated protocols not practicable manually to be designed, but at the present time lack of theoretical support prevents the full use of this option.

### Acknowledgements

The authors wish to thank M. Dupuy, A. Foucard, P. Meunier and E. Scohy for their help in the design and the construction of the set, and Professor Guetté for valuable discussions.

### References

1. Box, G. E. P. and Wilson, K. B., *J. Roy. Stat. B*, **XIII** (1951), 1.
2. Box, G. E. P. and Draper, N. R. *Evolutionary Operation* (John Wiley & Sons, 1969 ).
3. Winicov, H., Schainbaum, J., Buckley, J., Longino, G., Hill, J. and Berkoff, C. E., *Analytica Chimica Acta*, **103** (1978), 469.
4. Chodosh, D. F., Wdzieckowski, F. E., Schainbaum, J. and Berkoff, C. E., *Journal of Automatic Chemistry*, **5** (1983), 9.
5. Chodosh, D. F., Levinson, S. H., Weber, J. L., Kanholz, K. and Berkoff, C. E., *Journal of Automatic Chemistry*, **5** (1983), 103.
6. Kemp, M. G., *Chemistry and Industry*, **335** (1983).
7. Legrand, M., Foucard, A., *Journal of Chemical Education*, **55** (1978), 767.

### ASTM Task Group

A new ASTM (American Society for Testing and Materials) task group on diode array spectrophotometers is planned by Subcommittee E13.01 on Ultraviolet and Visible Spectroscopy, a branch of Committee E-13 on Molecular Spectroscopy. The organizational meeting will be held 25 February 1985, 7.00 p.m., at the Pittsburgh Conference & Exposition in New Orleans. Defining and establishing test methods and specifications for diode array spectrophotometers will be highlighted. Ultraviolet and visible spectrophotometer manufacturers and users are encouraged to participate in this standards writing activity. Committee E-13 will also hold its standards development meetings 25–27 February at the Pittsburgh Conference.
*More information from Dr Dianna G. Jones, Tracer Northern, 2551 W. Beltline Highway, Middleton, Wisconsin 53562, USA. Tel.: 608 831 6511.*

